# Automatic dental age estimation in adolescents via oral panoramic imaging

**DOI:** 10.3389/fdmed.2025.1618246

**Published:** 2025-06-26

**Authors:** Ze Li, Ning Xiao, Xiaoru Nan, Kejian Chen, Yingjiao Zhao, Shaobo Wang, Xiangjie Guo, Cairong Gao

**Affiliations:** ^1^ School of Forensic Medicine, Shanxi Medical University, Taiyuan, China; ^2^ Department of Orthodontics, Shanxi Provincial People’s Hospital, The Fifth Clinical Medical College of Shanxi Medical University, Taiyuan, China; ^3^ School of Information, Shanxi University of Finance and Economics, Taiyuan, China

**Keywords:** forensic dentistry, age estimation, pediatric dentistry, convolutional neural network, Demirjian method, oral panoramic imaging

## Abstract

**Object:**

In forensic dentistry, dental age estimation assists experts in determining the age of victims or suspects, which is vital for legal responsibility and sentencing. The traditional Demirjian method assesses the development of seven mandibular teeth in pediatric dentistry, but it is time-consuming and relies heavily on subjective judgment.

**Methods:**

This study constructed a largescale panoramic dental image dataset and applied various convolutional neural network (CNN) models for automated age estimation.

**Results:**

Model performance was evaluated using loss curves, residual histograms, and normal PP plots. Age prediction models were built separately for the total, female, and male samples. The best models yielded mean absolute errors of 1.24, 1.28, and 1.15 years, respectively.

**Discussion:**

These findings confirm the effectiveness of deep learning models in dental age estimation, particularly among northern Chinese adolescents.

## 1 Introduction

Age estimation plays a crucial role in various fields, including forensic science, orthodontics, pediatric healthcare, and social management, especially in pediatric dentistry (1, 2). Accurately determining an individual's physiological age is not only essential for forensic identification, personal identification, and criminal responsibility assessment but also directly impacts the evaluation of children's growth and development as well as the formulation of orthodontic treatment plans (3, 4).

Traditional dental age estimation methods rely on manually scoring the developmental stages of teeth and then estimating dental age using preestablished conversion tables, such as Demirjian method (5). While these methods can effectively reflect dental development within certain age ranges, their accuracy is influenced by factors such as population ethnicity, environmental conditions, nutritional status, and individual developmental differences. Due to the complex nonlinear relationships between tooth mineralization and root development, traditional methods struggle to fully capture these subtle variations. In particular, during late adolescence, as dental development approaches maturity, traditional methods often exhibit a “ceiling effect,” leading to significant prediction deviations and limiting their practical applicability (6).

With advancements in science and technology, new dental age estimation methods have emerged, incorporating medical imaging analysis and deep learning, especially convolutional neural network. Compared to traditional methods, convolutional neural network (CNNs) can capture subtle changes in dental microstructures more precisely, reduce human errors, and improve both accuracy and applicability. With the integration of intelligent algorithms, dental age estimation becomes even more precise and universally applicable, providing a more scientific basis for forensic identification, dental medicine, and child health assessment (7–9).

Dental radiographs serve as crucial imaging data for assessing the growth and development of children and adolescents, containing abundant information on dental development (10). Convolutional Neural Networks, has demonstrated exceptional performance in various fields such as medical diagnosis and pathological detection, thanks to its ability to automate feature extraction and represent multilevel information (11, 12). This technology offers a novel approach to automatic dental age estimation, overcoming the limitations of traditional methods and improving both accuracy and efficiency.

This study aims to explore an automatic dental age estimation method based on deep learning. The primary objective is to develop and optimize deep neural network models to automatically extract dental developmental features from panoramic dental radiographs, thereby achieving high precision age prediction. To accomplish this, the study employs various classic and advanced deep learning architectures, including LeNet5, AlexNet, VGG16, ResNet50, ConvNeXt, and Swin Transformer, and compares their performance in the dental age estimation task.

## 2 Materials and methods

### 2.1 Materials

This study selected a total of 3,790 panoramic dental radiographs (orthopantomograms) from children and adolescents aged 5–23 years who visited the Department of Dentistry at the Affiliated People's Hospital of the Medical University between June 2021 and December 2024. Among them, 1,693 were male, and 2,097 were female.

All imaging data used in this study were obtained through retrospective analysis and were not associated with any commercial interests. During the research process, the images were anonymized, with only gender, imaging date, and birthdate recorded to ensure the protection of personal privacy. According to the current Ethical Review Measures for Biomedical Research Involving Humans, this study is exempt from the requirement of obtaining informed consent.

Inclusion Criteria: (1) The sample consists of Han Chinese individuals who were born and have lived long-term in the North China region to ensure consistency in regional and ethnic characteristics. (2) The collected panoramic images must be clear and free of blurring, with the width difference between the left and right first permanent molar crowns not exceeding 20% to ensure measurement accuracy. (3) The sample must have a complete dentition with normal dental growth and development, without cavities, periodontal disease, dental trauma, or congenital or acquired tooth loss.

Exclusion Criteria: (1) Diseases affecting normal jawbone development, such as temporomandibular joint ankylosis, cleft lip and palate, jaw deformities, or jaw tumors. (2) History of maxillofacial trauma. (3) Previous orthodontic treatment. (4) Abnormal development of the third molar, such as short roots, malformed roots, or impacted third molars. (5) Individuals with chronic diseases, systemic conditions, genetic disorders, or abnormal physical development.

The distribution of gender characteristics and age characteristics of the samples involved in this study are shown in Tables 1, 2, respectively.

**Table 1:** Gender characteristics of the research subjects included.

Group	Number of people	Constituent ratio
Male	1,693	44.67%
Female	2,097	55.33%

**Table 2:** Age characteristics of the research subjects included.

Group	Number of people	Constituent ratio
Under 5 years old	169	4.46%
6–11 years old	2,284	60.26%
12–18 years old	918	24.22%
18 years old and above	419	11.06%

In the sample of this study, males accounted for 44.67% and females accounted for 55.33%, indicating a relatively balanced gender ratio with little deviation. However, considering the significant differences in tooth age development between genders, gender factors have a strong impact on tooth age estimation. Therefore, when constructing a dental age prediction model, it is necessary to perform gender stratified analysis on the samples to improve the model's prediction accuracy and generalization ability.

The age distribution of the sample shows a clear concentration trend, especially during the tooth replacement period (6–12 years old), with a sample proportion of as high as 60.26% in this age group. At this stage, the mineralization, eruption, and root development characteristics of teeth are relatively active and significant, which is a critical period for tooth age assessment research. In contrast, the proportion of samples in the age groups of under 5 years old (4.46%) and 18 years old and above (11.06%) is relatively low, which may be related to the lower frequency of individual visits or atypical and unrepresentative dental development characteristics in these stages.

**Figure 1:**
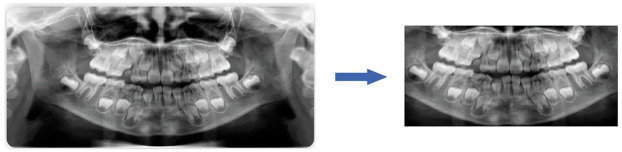
The preprocessing of panoramic dental images.

To maximize the use of image information from panoramic radiographs, irrelevant elements were first removed prior to analysis, ensuring that the images processed by the convolutional neural networks (CNNs) contained only the dental arch region, as illustrated in Figure 1.

**Figure 2:**
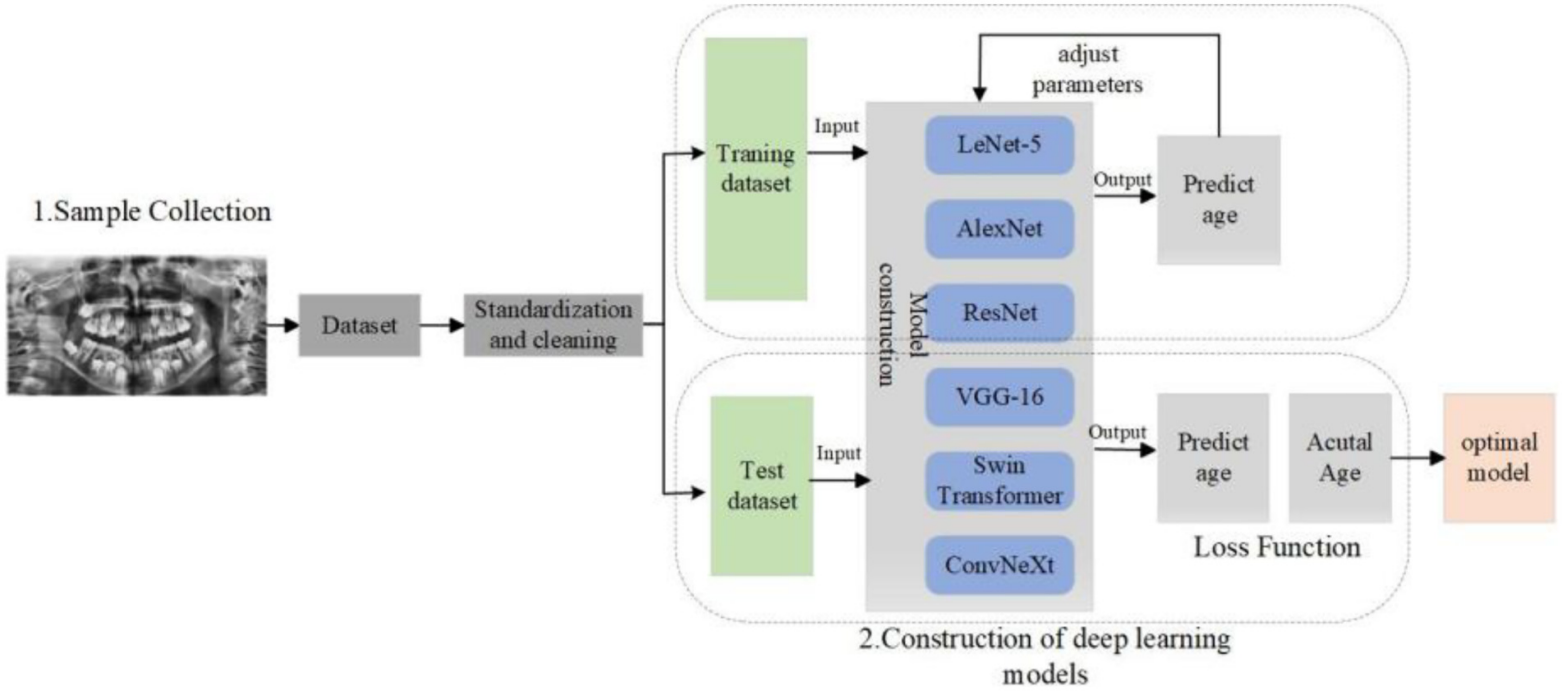
The flow chart of tooth age estimation based on deep learning.

The fully automated dental age estimation method involves the following steps: First, OpenCV image processing tools were used to locate and label the oral region in the radiographs (13). Second, various convolutional neural network models were employed to train on the panoramic dental images for age prediction. Finally, the trained CNN models were evaluated on a test set to assess their performance and to perform dental age estimation, as shown in the Figure 2.

### 2.2 Deep learning model and training

**Figure 3:**
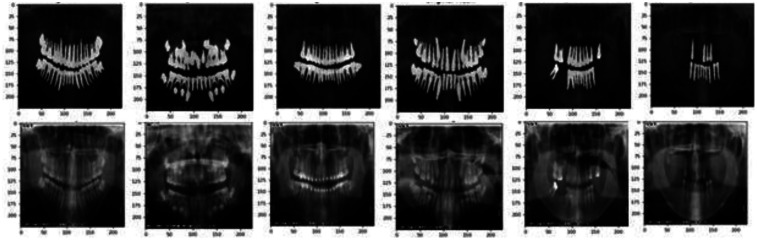
The result of tooth segmentation.

Automatic dental age estimation is a fine-grained visual regression task, made particularly challenging by the subtle inherent differences between individual teeth. Unlike traditional regression problems, this task requires precise recognition of fine visual cues. Motivated by the hypothesis that incorporating segmentation tasks can enhance regression performance, this study first compared various tooth segmentation methods and ultimately employed the U-Net architecture for tooth segmentation (14). Using the U-Net model, segmentation masks were automatically generated for the teeth in panoramic radiographs. These masks were then manually reviewed to ensure that they accurately captured the complete tooth regions, as shown in Figure 3. After verifying the masks, various deep learning models were applied to perform age prediction based on the segmented images. The models used include LeNet-5, AlexNet, VGG-16, ResNet-50, Swin Transformer, and ConvNeXt (15).

The following subsection will introduce the specific architectures of these neural network models.

#### 2.2.1 LeNet-5

The LeNet-5 network is a classical convolutional neural network (CNN) architecture, known for its simplicity and effectiveness in early image recognition tasks (16). Its main features include the use of alternating convolutional and pooling layers to progressively extract features, followed by fully connected layers for classification. Through weight sharing and local connections, LeNet-5 significantly reduces the computational load while preserving spatial information.

By combining convolution and pooling operations, LeNet-5 effectively captures both local and global features in the image. This structure not only reduces the number of parameters but also lowers computational cost and minimizes the risk of overfitting, making it a suitable choice for tasks involving limited data or relatively simple image structures.

#### 2.2.2 AlexNet

AlexNet builds upon the foundation laid by LeNet and introduces several key innovations that significantly improve the performance of convolutional neural networks, especially on large-scale image classification tasks (17). One major advancement is the introduction of the ReLU (Rectified Linear Unit) activation function. ReLU effectively alleviates the vanishing gradient problem, speeds up training, and is computationally efficient. Its operation is simple: when the input is greater than zero, the output equals the input; when the input is less than or equal to zero, the output is zero.

However, a drawback of ReLU is that neurons can “die” during training if they fall into the negative range and stop updating, which may reduce model capacity. To address model overfitting and improve generalization, AlexNet also incorporates regularization techniques. One of the most notable is Dropout, which randomly deactivates a subset of neurons during training with a certain probability. These deactivated neurons do not participate in forward or backward propagation for that iteration.

#### 2.2.3 VGG-16

VGG-16 is a classic model from the VGG architecture family, known for its deep network structure and simple yet powerful design (18). Its main strength lies in its ability to extract rich, multi-level features from images through a significantly deep architecture. The name “VGG-16” comes from its total of 16 weight layers, which include 13 convolutional layers and 3 fully connected layers. One of the defining features of VGG-16 is its architectural consistency and scalability. All convolutional layers use uniform 3 × 3 kernels, which helps capture fine-grained spatial features while keeping the design straightforward. Additionally, a 2 × 2 max pooling layer is inserted after every two convolutional layers to reduce spatial dimensions while retaining important information.

This uniform structure not only simplifies network design and implementation but also enhances training stability and computational efficiency. Due to its balance between depth and simplicity, VGG-16 has become a foundational model for many computer vision tasks and is widely used as a baseline in both academic research and practical applications.

#### 2.2.4 ResNet-50

ResNet-50 introduces the concept of residual learning, which allows each block in the network to learn the residual between the input and the desired output, rather than attempting to learn a direct and potentially complex mapping (19). This innovation effectively addresses the vanishing gradient and performance degradation problems that typically occur when the depth of a neural network increases.

In traditional deep networks, each layer attempts to learn a complete transformation from input to output. However, as networks become deeper, this task becomes increasingly difficult, leading to training challenges and a drop in accuracy. ResNet-50 overcomes this by adding shortcut connections, which bypass one or more layers and allow the network to learn residual functions more easily.

#### 2.2.5 Swin transformer

Swin Transformer uses the Transformer architecture to process image data and effectively improves computational efficiency and modelling capability through local window partitioning and shifted window techniques (20). The Swin Transformer consists of multiple stages, where the size of the output feature maps gradually decreases, while the number of channels increases across stages. This design helps in extracting multi-scale image features.

By utilizing two core components—Window-based Multi-Head Self-Attention (W-MSA) and Shifted Window-based Multi-Head Self-Attention (SW-MSA)—the model reduces computational load while capturing local features. Swin Transformer divides the input feature map into several non-overlapping windows. Within each window, the multi-head self-attention mechanism computes queries, keys, and values. Through this mechanism, the model captures feature relationships from different perspectives. Finally, the outputs from all heads are concatenated and passed through a linear transformation to produce the final output.

#### 2.2.6 ConvNeXt

ConvNeXt draws inspiration from Transformer design principles to enhance the performance of traditional convolutional neural networks (CNNs) (21). Each stage of ConvNeXt consists of a series of convolutional and pooling layers that progressively reduce the resolution of the feature maps while increasing the number of channels, allowing the model to extract features at multiple scales.

ConvNeXt adopts relatively large convolutional kernels to capture broader contextual information in images. It also leverages a combination of depth-wise convolutions and pointwise (1 × 1) convolutions, which increases the network's expressive power while maintaining computational efficiency.

## 3 Results

### 3.1 Implantation details and evaluation metrix

To expand the dataset and enhance model robustness, data augmentation techniques such as image rotation and flipping were applied to the original images. Before validation, this study applied data augmentation techniques (horizontal flip, vertical flip, rotation, and Gaussian blur.) to expand the original set of 3,790 images to 18,950 images (22, 23). These augmented images were then split into training, validation, and test sets in a 7:2:1 ratio. As a result, the dataset contained 13,265 training images, 3,790 test images, and 1,895 validation images. All experiments were conducted on a workstation equipped with an NVIDIA RTX 3090 (32GB) GPU running CUDA 11.4. The models were developed using Python 3.11 and PyTorch v1.12.1.

To prevent data leakage, all augmented versions of a given original image were grouped and assigned to the same dataset split. Stratification and splitting were performed prior to data augmentation, ensuring no overlap of source images across training, validation, and test sets. Additionally, when applicable, splits were made at the patient level to avoid intra-subject information leakage.

During model evaluation, this study calculated Accuracy, Mean Absolute Error (MAE), and the Coefficient of Determination (*R*^2^). By comparing the performance metrics of different models, the most optimal model for dental age estimation was selected. The calculation formulas for different performance metrics are shown in Equations 1–3:

(1) $$MAE=\frac{1}{n}\sum_{i=1}^{n}\left|-{\hat{y}}_{i}\right|$$

(2) $$R^{2}=1-\frac{1}{n}\sum_{i=1}^{n}{\left(y_{i}-{\hat{y}}_{i}\right)}^{2}/\sum_{i=1}^{n}{\left(y_{i}-\bar{y}\right)}^{2}$$

(3) $$Accuracy=\frac{TP}{TP+FP}$$

In the above formula, $y_{i}\;$ is the true age of the i-th sample, ${\hat{y}}_{i}$ is the predicted age of the i-th sample, $y_{i}\;$ is the average of the true ages of the samples, and *n* is the sample size. TP is the correct sample for prediction (the residual between the predicted value and the true value within 1 year old). FP is the wrong sample for prediction. This research discretized continuous age values into 1-year bins to assess performance from a clinically relevant perspective. In dental age estimation, a prediction within ±1 year of the actual age is generally acceptable in practice. Therefore, we defined predictions within this range as “correct” and used this criterion to calculate an accuracy metric.

### 3.2 Loss function and hyperparameter

By analyzing the data samples collected in this study, we observed a class imbalance phenomenon, with most samples concentrated in the 6–18 age range. Therefore, when designing the loss functions for the various network models, it is important to consider not only model performance but also the issue of class imbalance. In this study, we followed approaches reported in the literature and adopted the balanced MSE loss as the loss function for all network models. The formula for the loss function is shown in Equation 4.

(4) $$L=\frac{1}{N}\sum_{i=1}^{N}{\left(y_{i}-{\hat{y}}_{i}\right)}^{2}\cdot w_{i}$$

Here, $w_{i}$ represents the weight assigned to each class, which is used to suppress the influence of dominant classes and increase the relative importance of minority classes. Specifically, $w_{i}$ is defined as the inverse of the frequency $f_{i}$ of the class to which sample iii belongs, i.e., $w_{i}=1/f_{i}$.

Hyperparameters are parameters that must be set before training a deep learning model. They control the model's architecture and learning process, such as the learning rate, optimizer, and weight decay. In this study, the optimal hyperparameters for each network model are listed in Table 3.

**Table 3:** The optimal hyperparameters of convolutional network used in this article.

Network model	Learning rate	Optimizer	Epoch	Weight decay
LeNet-5	0.01	RMSProp	15	5 × 10^−5^
AlexNet	0.0005	Adam	25	5 × 10^−5^
VGG-16	0.0008	Adadelta	22	1 × 10^−4^
ResNet-50	0.001	Adam	20	1 × 10^−5^
Swin Transformer	0.0003	AdamW	30	1 × 10^−5^
ConvNeXt	0.0015	Adagrad	18	1 × 10^−4^

### 3.3 Comparison of tooth segmentation

Tooth segmentation can significantly enhance the accuracy and robustness of dental age estimation by isolating individual teeth or tooth structures—from irrelevant background information. In this study, incorporating a segmentation step prior to age estimation helped the model learn more discriminative patterns, ultimately contributing to improved regression performance. This study first compared various tooth segmentation methods, including dice coefficients, segmentation time. The performance of different segmentation methods in tooth segmentation as shown in Table 4.

**Table 4:** The performance of different segmentation in tooth segmentation.

Segmentation method	Dice coefficient	Segmentation time(s)
FCN	78.70	4.31
DeepLab v1	81.45	5.12
SegNet	88.71	5.08
U-Net	91.04	4.93

The DICE coefficient is an indicator used to measure the effectiveness of image segmentation, with higher values indicating better segmentation performance. As seen in Table 4, U-Net performs significantly better than the other methods in terms of segmentation quality. Although its segmentation time is slightly longer (4.93 s), overall, it provides the best performance. Therefore, this research ultimately employed the U-Net architecture for tooth segmentation due to its effectiveness and robustness.

### 3.4 Comparison of quantitative results

To evaluate the effectiveness of convolutional neural networks (CNNs) in dental age estimation, the study first compared the performance of different CNN architectures based on various metrics, as shown in Table 5.

**Table 5:** Performance indicators of different network models.

Network model	MAE	*R* ^2^	Accuracy
LeNet-5	3.85	0.682	0.609
AlexNet	3.65	0.741	0.663
VGG-16	4.13	0.722	0.723
ResNet-50	1.13	**0.924**	**0.839**
Swin Transformer	3.87	0.672	0.629
ConvNeXt	**1.12**	0.918	0.820

The bold values means the optimal value.

Table 5 shows the performance metrics of six different network models in the dental age estimation task, including Mean Absolute Error, Coefficient of Determination, and Accuracy. Lower MAE values, higher *R*^2^ values, and higher accuracy indicate better model performance (24).

### 3.5 Comparison of loss function

The loss function curve is a vital tool for monitoring the training dynamics of deep learning models (25). It illustrates how the loss value changes over training epochs, enabling researchers to determine whether the model is converging, overfitting, or underfitting.

**Figure 4:**
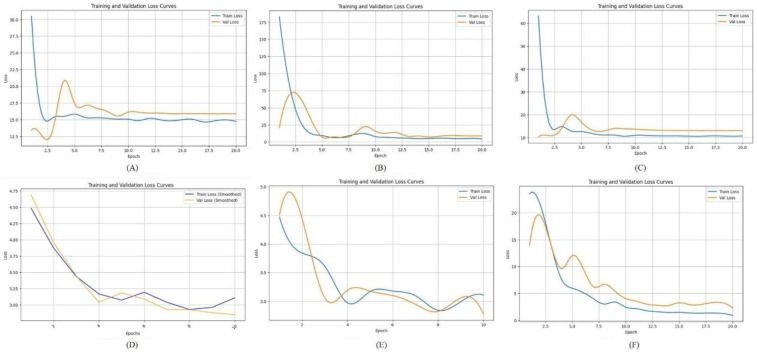
The loss function of different deep learning model. **(A)** LeNet-5; **(B)** AlexNet; **(C)** VGG-16; **(D)** ResNet-50; **(E)** Swin Transformer; **(F)** ConvNeXt.

In this study, we compared the loss curves of multiple convolutional neural network architectures throughout the training process, as shown in Figure 4. By observing the trend and stability of these curves, we can assess each model's learning efficiency and generalization capability. A well-performing model typically demonstrates a smooth and steadily declining loss curve on the training set, accompanied by a similarly stable or slightly fluctuating validation loss curve.

Figure 4A shows the loss function curve of the LeNet-5 model during training. Both the training loss and validation loss exhibit a sharp decline during the initial 1–2 epochs, dropping rapidly from a high starting point of approximately 60–70 down to a range of 10–15. However, after this initial descent, the loss values do not continue to decrease significantly. Figure 4B illustrates the loss function curve of the AlexNet model. As shown in the figure, the validation loss initially decreases in tandem with the training loss and reaches a relatively low point around epochs 2–3. Afterward, it experiences slight fluctuations but gradually stabilizes at a level close to the training loss. This suggests that no significant overfitting or divergence occurred in the later stages of training, indicating relatively stable learning behavior. Figure 4C displays the loss curve of VGG-16. In this case, the training loss remains slightly lower than the validation loss throughout the training process. However, the gap between the two curves is not substantial, suggesting that VGG-16 maintains a balanced generalization ability. This implies that the model does not suffer from pronounced overfitting or instability during training, and it performs consistently across both training and validation datasets. Figure 4D shows the loss trajectory of ResNet-50. During the early training phase, both the training and validation losses drop rapidly from an initial high value (approximately 4.75) to a range between 2.5 and 3.0. This indicates that the model quickly learns low-level or general image features. In the later training stages, the training loss remains slightly lower than the validation loss, but the margin is narrow, which reflects good model fitting and generalization performance on both datasets. Figure 4E presents the loss curve of the Swin Transformer model. Compared to other deep networks such as ResNet-50, the Swin Transformer exhibits a relatively high validation loss throughout training and fails to converge to a lower and stable value. This trend suggests that the model faces challenges in learning effectively from the current dataset, potentially due to the limited sample size, insufficient tuning, or the architecture being less compatible with the specific characteristics of panoramic dental images. Figure 4F illustrates the loss curve of ConvNeXt. In this case, the training loss consistently stays slightly below the validation loss, with no pronounced divergence. This pattern indicates that the model does not exhibit severe overfitting or underfitting in the later training epochs. The overall trend suggests that ConvNeXt achieves a well-balanced fit between the training and validation sets, demonstrating strong learning stability and generalization capability.

### 3.6 Standardized residual histogram and normal P-P plot of standardized residuals

To evaluate the distribution of prediction errors and the reliability of the regression model, this study employed two key diagnostic tools: the Standardized Residual Histogram (SRH) and the normal P–P plot of standardized residuals (26).

The standardized residual histogram visualizes the distribution of residuals. A bell-shaped, symmetric histogram indicates that residuals are approximately normally distributed, which supports one of the core assumptions in linear regression models. A skewed or multimodal distribution, however, might suggest model misspecification, outliers, or non-linearity in the data.

The normal probability–probability (P–P) plot of standardized residuals further tests the normality assumption by plotting the cumulative distribution of the observed standardized residuals against a theoretical normal distribution. If the residuals are normally distributed, the points on the P–P plot should fall closely along the 45-degree reference line.

**Figure 5:**
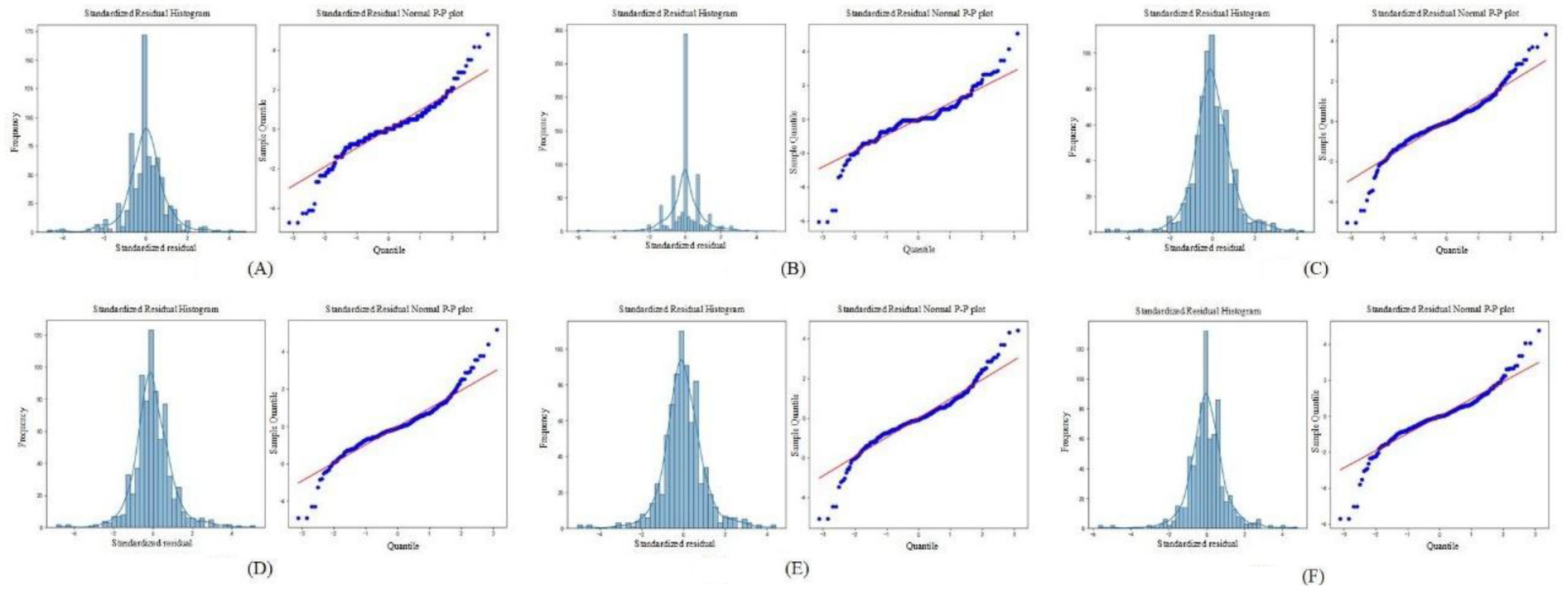
The standardized residual histogram and normal P-P plot of standardized residual for different models. **(A)** LeNet-5; **(B)** AlexNet; **(C)** VGG-16; **(D)** ResNet-50; **(E)** Swin Transformer; **(F)** ConvNeXt.

The Standardized Residual Histogram and Normal P-P Plot of Standardized Residual for different models are shown in the Figure 5.

As shown in Figure 5, the residuals from all deep learning models approximately follow a normal distribution. This is evidenced by the standardized residual histograms, which exhibit a bell-shaped curve, and the normal P-P plots, in which most of the data points lie close to the diagonal reference line. These visual assessments suggest that the residuals satisfy the assumption of normality. Furthermore, based on the residual statistics, although a few outliers are observed, the overall standard deviation of the residuals remains relatively small. This implies that prediction errors are generally limited in magnitude, and the deep learning models are not significantly biased. Taken together, the conformity of the residuals to a normal distribution, the low dispersion, and the limited number of outliers validate the appropriateness and robustness of the deep learning models for dental age estimation tasks. These results support the conclusion that the models exhibit good predictive performance and reliability on the given dataset.

### 3.7 Evaluation of the optimal age estimation model

The optimal age estimation models for the total sample, as well as for the female and male subgroups, were identified. The performance evaluation across different age ranges is detailed in Tables 5–7. The residual value in the Tables 6–8 is the residual between the true value and the predicted value.

**Table 6:** The predictive performance of the total sample optimal age estimation model (ResNet-50).

Age range	Number of samples	True value	Predicted value	Residual	MAE
3.00–3.99	7	3.00 ± 0.00	4.25 ± 0.00	−1.25 ± 0.00	1.2500
4.00–4.99	32	4.00 ± 0.00	4.67 ± 0.31	−0.67 ± 0.31	0.6666
5.00–5.99	143	5.00 ± 0.00	5.81 ± 1.95	−0.81 ± 1.95	0.9351
6.00–6.99	196	6.00 ± 0.00	6.67 ± 1.57	−0.67 ± 1.57	0.9038
7.00–7.99	260	7.00 ± 0.00	7.16 ± 0.84	−0.16 ± 0.84	0.5471
8.00–8.99	471	8.00 ± 0.00	8.24 ± 0.87	−0.24 ± 0.87	0.6236
9.00–9.99	236	9.00 ± 0.00	9.30 ± 1.25	−0.30 ± 1.25	0.8802
10.00–10.99	421	10.00 ± 0.00	10.27 ± 1.40	−0.27 ± 1.40	0.9226
11.00–11.99	316	11.00 ± 0.00	11.25 ± 1.10	−0.25 ± 1.10	0.8104
12.00–12.99	375	12.00 ± 0.00	12.47 ± 1.55	−0.47 ± 1.55	1.1396
13.00–13.99	216	13.00 ± 0.00	13.16 ± 1.39	−0.16 ± 1.39	1.1277
14.00–14.99	212	14.00 ± 0.00	14.16 ± 1.52	−0.16 ± 1.52	1.1590
15.00–15.99	172	15.00 ± 0.00	15.12 ± 1.70	−0.12 ± 1.70	1.3214
16.00–16.99	161	16.00 ± 0.00	15.54 ± 1.74	0.46 ± 1.74	1.4765
17.00–17.99	140	17.00 ± 0.00	15.87 ± 2.18	1.13 ± 2.18	2.0089
18.00–18.99	132	18.00 ± 0.00	17.16 ± 2.06	0.84 ± 2.06	1.6634
19.00–19.99	126	19.00 ± 0.00	17.49 ± 2.05	1.51 ± 2.05	1.7900
20.00–20.99	96	20.00 ± 0.00	18.69 ± 1.34	1.31 ± 1.34	1.3106
21.00–21.99	62	21.00 ± 0.00	19.79 ± 1.28	1.21 ± 1.28	1.2161
22.00–22.99	15	22.00 ± 0.00	19.91 ± 1.44	2.09 ± 1.44	1.6182
23.00–23.99	1	23.00 ± 0.00	21.50 ± 0.00	1.50 ± 0.00	1.5000

**Table 7:** The predictive performance of the female sample optimal age estimation model (ResNet-50).

Age range	Number of samples	True value	Predicted value	Residual	MAE
3.00–3.99	5	3.00 ± 0.00	5.22 ± 0.00	−2.22 ± 0.00	2.2200
4.00–4.99	21	4.00 ± 0.00	6.50 ± 2.21	−2.50 ± 2.21	2.4975
5.00–5.99	78	5.00 ± 0.00	6.82 ± 2.33	−1.82 ± 2.33	1.8226
6.00–6.99	117	6.00 ± 0.00	6.92 ± 1.50	−0.92 ± 1.50	0.9670
7.00–7.99	139	7.00 ± 0.00	7.13 ± 0.49	−0.13 ± 0.49	0.3618
8.00–8.99	238	8.00 ± 0.00	8.20 ± 0.77	−0.20 ± 0.77	0.5864
9.00–9.99	129	9.00 ± 0.00	9.54 ± 1.61	−0.54 ± 1.61	0.9619
10.00–10.99	264	10.00 ± 0.00	10.47 ± 1.51	−0.47 ± 1.51	1.0107
11.00–11.99	169	11.00 ± 0.00	11.66 ± 1.22	−0.66 ± 1.22	0.9887
12.00–12.99	221	12.00 ± 0.00	12.46 ± 1.79	−0.46 ± 1.79	1.3938
13.00–13.99	129	13.00 ± 0.00	13.68 ± 1.44	−0.68 ± 1.44	1.4048
14.00–14.99	96	14.00 ± 0.00	14.51 ± 1.55	−0.51 ± 1.55	1.4750
15.00–15.99	102	15.00 ± 0.00	15.28 ± 1.46	−0.28 ± 1.46	1.3165
16.00–16.99	87	16.00 ± 0.00	15.43 ± 1.33	0.57 ± 1.33	1.1094
17.00–17.99	85	17.00 ± 0.00	15.63 ± 1.75	1.37 ± 1.75	1.6894
18.00–18.99	89	18.00 ± 0.00	16.89 ± 1.89	1.11 ± 1.89	1.5605
19.00–19.99	64	19.00 ± 0.00	16.45 ± 2.01	2.55 ± 2.01	2.6138
20.00–20.99	28	20.00 ± 0.00	17.98 ± 1.82	2.02 ± 1.82	2.0200
21.00–21.99	26	21.00 ± 0.00	19.02 ± 1.10	1.88 ± 1.10	1.9134
22.00–22.99	10	22.00 ± 0.00	20.18 ± 1.31	1.82 ± 1.31	1.8617

**Table 8:** The predictive performance of the male sample optimal age estimation model (ConvNeXt).

Age range	Number of samples	True value	Predicted value	Residual	MAE
3.00–3.99	2	3.00 ± 0.00	5.22 ± 0.00	−2.22 ± 0.00	2.2200
4.00–4.99	11	4.00 ± 0.00	5.42 ± 0.10	−1.42 ± 0.10	1.4212
5.00–5.99	65	5.00 ± 0.00	5.53 ± 0.34	−0.53 ± 0.34	0.5393
6.00–6.99	79	6.00 ± 0.00	6.88 ± 1.83	−0.88 ± 1.83	1.1239
7.00–7.99	121	7.00 ± 0.00	7.37 ± 0.82	−0.37 ± 0.82	0.6075
8.00–8.99	233	8.00 ± 0.00	8.21 ± 0.64	−0.21 ± 0.64	0.5507
9.00–9.99	107	9.00 ± 0.00	9.19 ± 0.81	−0.19 ± 0.81	0.7343
10.00–10.99	157	10.00 ± 0.00	10.12 ± 0.88	−0.12 ± 0.88	0.6903
11.00–11.99	147	11.00 ± 0.00	11.35 ± 1.22	−0.35 ± 1.22	1.0379
12.00–12.99	154	12.00 ± 0.00	12.46 ± 1.21	−0.46 ± 1.21	1.0107
13.00–13.99	87	13.00 ± 0.00	12.80 ± 0.75	0.19 ± 0.75	0.5561
14.00–14.99	116	14.00 ± 0.00	13.92 ± 0.97	0.07 ± 0.97	0.7000
15.00–15.99	70	15.00 ± 0.00	14.28 ± 1.56	0.71 ± 1.56	1.3713
16.00–16.99	74	16.00 ± 0.00	15.27 ± 1.73	0.72 ± 1.73	1.6517
17.00–17.99	55	17.00 ± 0.00	16.81 ± 1.38	0.18 ± 1.38	1.2642
18.00–18.99	43	18.00 ± 0.00	18.09 ± 1.17	−0.09 ± 1.17	0.9928
19.00–19.99	62	19.00 ± 0.00	17.83 ± 2.03	1.16 ± 2.03	1.4387
20.00–20.99	68	20.00 ± 0.00	18.74 ± 0.93	1.25 ± 0.93	1.2541
20.00–20.99	36	21.00 ± 0.00	19.37 ± 1.36	1.63 ± 1.36	1.5217
21.00–21.99	5	22.00 ± 0.00	20.16 ± 1.23	1.84 ± 1.23	1.7638
23.00–23.99	1	23.00 ± 0.00	21.46 ± 0.00	1.54 ± 0.00	1.5385

According to Tables 6–8, it is evident that the optimal age estimation models demonstrate varying levels of accuracy across different age groups. The models exhibit relatively high accuracy in the adolescent population aged between 5.00 and 15.99 years, with the mean absolute error approaching approximately 1 year. However, for individuals aged 16 years and above, the accuracy of age estimation gradually declines. Specifically, the MAE values for the total sample in the 4.00–11.99 age group are all under 1 year, indicating strong performance. Similarly, the MAE values for the female subgroup in the 6.00–9.99 and 11.00–11.99 age intervals are below 1 year. In the male subgroup, the model achieved an MAE close to 1 year across the 5.00–15.99 age range.

These findings suggest that combining machine learning techniques with Demirjian's method yields promising results in estimating dental age among children and adolescents in North China—especially during early and middle childhood, where predictive performance is particularly robust. However, the model's performance significantly declines when applied to individuals over the age of 16.

Based on clinical observations and a review of relevant literature, this performance trend can be attributed to the developmental characteristics of the dentition. The age ranges from 6 to 15 represents a crucial period for dental development, during which the permanent dentition undergoes active eruption and replacement. During this stage, the morphological differentiation of tooth crowns and the calcification of roots follow a relatively consistent and predictable pattern. Imaging features such as dentin deposition and root canal closure undergo continuous and discernible changes, which are well-suited for quantitative feature extraction by machine learning algorithms.

In contrast, after the age of 15, tooth development slows, and growth patterns become increasingly influenced by individual variation, hormonal fluctuations during and after puberty, as well as environmental and pathological factors. These elements introduce greater heterogeneity and irregularity in dental development, making it more challenging for algorithms to learn stable predictive patterns. Consequently, the model's performance in older adolescents and adults is less reliable.

### 3.8 Evaluation of the optimal age estimation model

**Figure 6:**
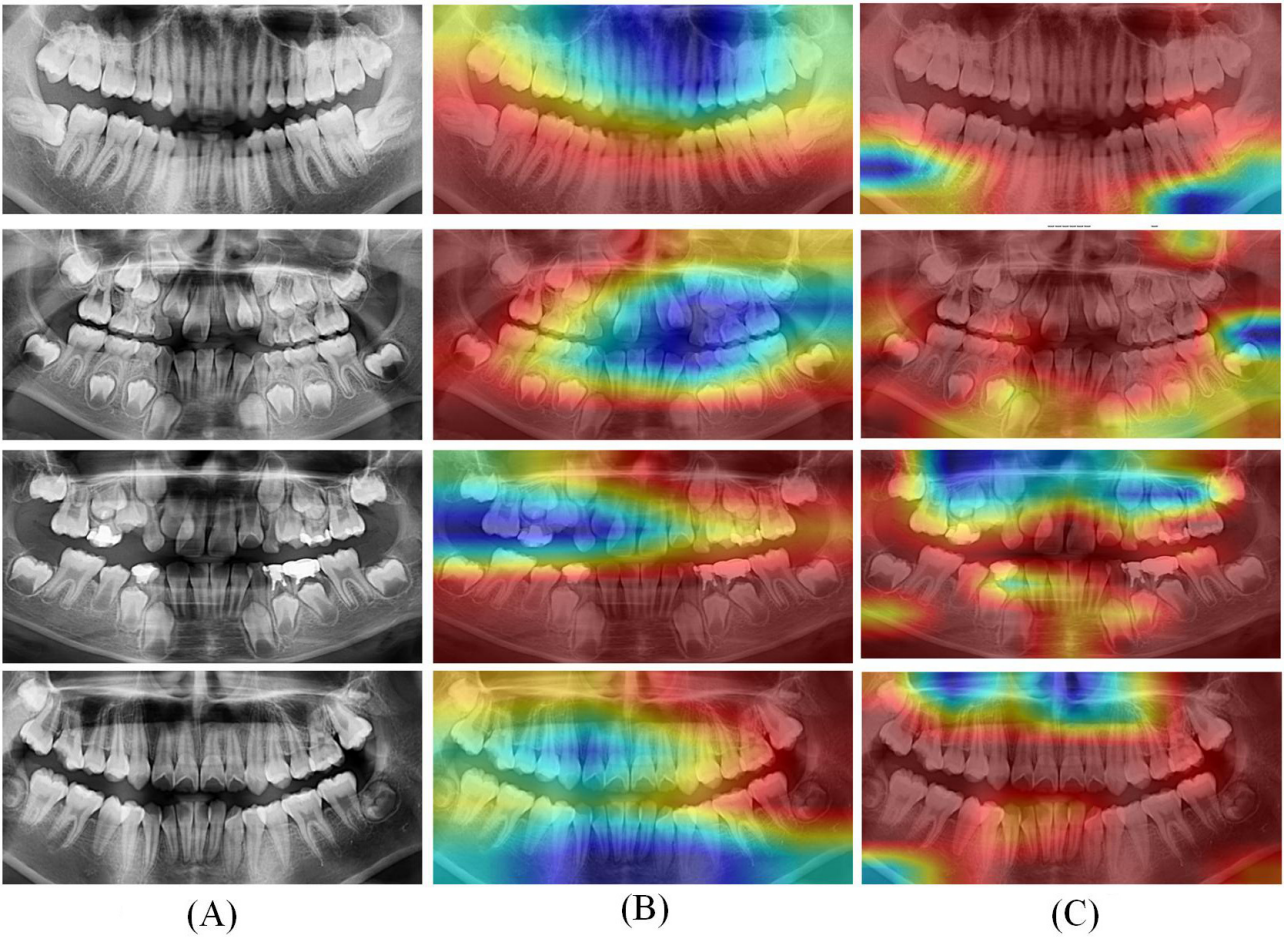
Grad CAM visualization results of optimal deep learning models. **(A)** Original CT; **(B)** Heatmaps of ResNet-50; **(C)** Heatmaps of ConvNeXt.

To visually demonstrate the effect, features with shapes of (416, 416, 32) were extracted from the ResNet-50 and ConvNeXt for visualization. The visualization results are shown in Figure 6.

In Figure 6, the left column (A) displays the original panoramic dental images, the column (B) presents the corresponding model-generated attention heatmaps of ResNet-50 and the column (C) presents the attention heatmaps of ConvNeXt. The patients in the first to fourth rows are aged 8, 11, 15, and 20, respectively. As illustrated in Figure 6, the model primarily focuses on the dental arch, capturing key features such as apical closure and occlusal surface wear. In children's patients, particularly those under the age of 10, the ResNet-50 pays closer attention to areas of permanent tooth eruption, root development, and deciduous tooth resorption. Observing from column C, the ConvNeXt focuses on the left posterior teeth and anterior mandible. These visualization results highlight the deep learning models strong biological interpretability and its ability to effectively distinguish age-related dental characteristics.

## 4 Discussion

Dental age estimation is a key technology widely used in various fields such as forensic medicine, clinical medicine, archaeology, and identification of minors (27, 28). The traditional method of inferring tooth age mainly relies on experienced dental experts to make judgments by observing the development and wear of teeth (29). However, this method is not only time-consuming, but also subjective, which may lead to biases in judgments between different experts. The traditional machine learning based dental age estimation method has improved the automation level to a certain extent, but its feature extraction process relies on manual experience and is difficult to fully explore the deep level information of the data. Adolescents in North China may be influenced by various factors such as genetics, environment, and dietary habits during tooth development, which may result in differences in the speed and morphological characteristics of tooth growth compared to adolescents in other regions (30). Therefore, using universal methods for inferring tooth age may not be well adapted to the specific growth patterns of adolescents in North China, while deep learning can extract tooth age features suitable for North China adolescents through large-scale regional data training, improving prediction accuracy.

The application of deep learning algorithms in the field of age estimation has become increasingly prevalent in recent years. This article proposes a deep learning-based model for automatically inferring tooth age and validates it on a collected dataset, demonstrating the significant importance and advantages of deep learning in the field of dental age estimation. This study conducted extensive training and validation of six deep learning architectures on a large-scale dental panoramic image dataset for automatic dental age estimation. The experimental results revealed marked differences in model performance across various evaluation metrics, including accuracy, stability, and convergence speed. Based on the Mean Absolute Error metrics evaluated on the training dataset, ResNet-50 demonstrated the best overall performance for both the total sample and the female subgroup, while ConvNeXt outperformed the other models in the male subgroup. These findings indicate the robustness and adaptability of different network architectures to varying population characteristics. Compared with traditional methods of dental age estimation—such as those based on manual scoring systems like Demirjian's method, which involve complex and time-consuming calculations and score conversions—deep learning models offer substantial advantages. Moreover, deep learning algorithms exhibit superior adaptability to data from diverse populations. This is particularly beneficial when handling datasets characterized by complex, nonlinear relationships, especially in the context of large-scale, population-based dental studies (31). As presented in the literature (32), the author used convolutional neural networks to diagnose dental age in 5,898 panoramic x-ray images. The results showed that compared to traditional methods, convolutional neural networks not only achieved satisfactory results, but also had faster inference speed, eliminating the need for additional learning from experts.

Although deep neural networks have significantly improved the overall accuracy of dental age estimation in this study, their predictive performance remains limited for older adolescents and adults aged 16 years and above. A noticeable tendency toward systematic underestimation and higher prediction errors was observed in this age group. This limitation is primarily attributed to the biological characteristics of dental development—specifically, the completion of crown and root formation, leading to reduced morphological variation and lower distinguishability in dental radiographs. Consequently, deep learning models struggle to extract informative features from these relatively static developmental stages. Moreover, the performance of models like ResNet-50 and ConvNeXt in this study heavily relies on the availability of large-scale, high-quality dental image datasets. Therefore, in the future, expanding the sample size for high-age and edge cases, improving the representativeness of the dataset, and optimizing data augmentation strategies are essential to enhance the generalizability and robustness of age estimation models in future applications.

In addition, due to current data access and regulatory restrictions, external datasets cannot be independently validated. However, considering the importance of evaluating the robustness of models for different populations, this study is actively seeking cooperation with other regions to establish a more heterogeneous, multi center dataset. In future work, this study will prioritize external validation of multi-ethnic and geographically diverse groups to ensure the wider applicability and fairness of the model.

## Data Availability

The raw data supporting the conclusions of this article will be made available by the authors, without undue reservation.

## References

[1] Shan W Sun Y Hu L Qiu J Huo M Zhang Z Boosting algorithm improves the accuracy of juvenile forensic dental age estimation in southern China population. Sci Rep. (2022) 12(1):15649. 10.1038/s41598-022-20034-9 36123377 PMC9485148

[2] Mohamed EG Redondo RPD Koura A EL-Mofty MS Kayed M. Dental age estimation using deep learning: a comparative survey. Computation. (2023) 11(2):18. 10.3390/computation11020018

[3] De Donno A Angrisani C Mele F Introna F Santoro V. Dental age estimation: Demirjian’s versus the other methods in different populations. A literature review. Med Sci Law. (2021) 61(1_suppl):125–9. 10.1177/0025802420934253 33591866

[4] Kiswanjaya B Taufiq SR Syahraini SI Yoshihara A. The influence of age, sex, and mandibular morphometric parameters on cortical bone width and erosion: a panoramic radiography study. Front Dent Med. (2025) 6:1558372. 10.3389/fdmed.2025.1558372 40212338 PMC11983699

[5] Melo M Ata-Ali F Ata-Ali J Martinez Gonzalez JM Cobo T. Demirjian and cameriere methods for age estimation in a Spanish sample of 1386 living subjects. Sci Rep. (2022) 12(1):2838. 10.1038/s41598-022-06917-x 35181746 PMC8857188

[6] Shen S Liu Z Wang J Fan L Ji F Tao J. Machine learning assisted cameriere method for dental age estimation. BMC Oral Health. (2021) 21(1):641. 10.1186/s12903-021-01996-0 34911516 PMC8672533

[7] Phulari RGS Dave EJ. Evolution of dental age estimation methods in adults over the years from occlusal wear to more sophisticated recent techniques. Egypt J Forensic Sci. (2021) 11:1–14. 10.1186/s41935-021-00250-633432273

[8] Milošević D Vodanović M Galić I Subašić M. Automated estimation of chronological age from panoramic dental x-ray images using deep learning. Expert Syst Appl. (2022) 189:116038. 10.1016/j.eswa.2021.116038

[9] Iruvuri AG Miryala G Khan Y Ramalingam NT Sevugaperumal B Soman M Revolutionizing dental imaging: a comprehensive study on the integration of artificial intelligence in dental and maxillofacial radiology. Cureus. (2023) 15(12):e50292. 10.7759/cureus.50292 38205468 PMC10776831

[10] Jundaeng J Chamchong R Nithikathkul C. Advanced AI-assisted panoramic radiograph analysis for periodontal prognostication and alveolar bone loss detection. Front Dent Med. (2025) 5:1509361. 10.3389/fdmed.2024.1509361 39917716 PMC11797906

[11] Ataş İ Özdemir C Ataş M Doğan Y. Forensic dental age estimation using modified deep learning neural network. Balkan J Electr Comput Eng. (2023) 11(4):298–305. 10.17694/bajece.1351546

[12] Almarghlani A Alsahafi RA Alqahtani FK Alnowailaty Y Barayan M Aladwani A Assessment of pulpal changes in periodontitis patients using CBCT: a volumetric analysis. Front Dent Med. (2025) 6:1549281. 10.3389/fdmed.2025.1549281 40612331 PMC12222284

[13] Gong S Kumar R Kumutha D. Design of lighting intelligent control system based on OpenCV image processing technology. Int J Uncertain Fuzziness Knowl-Based Syst. (2021) 29(Supp01):119–39. 10.1142/S0218488521400079

[14] Hou S Zhou T Liu Y Dang P Lu H Shi H. Teeth U-net: a segmentation model of dental panoramic x-ray images for context semantics and contrast enhancement. Comput Biol Med. (2023) 152:106296. 10.1016/j.compbiomed.2022.106296 36462370

[15] Kim YR Choi JH Ko J Jung Y-J Nam S-H Chang W-D. Age group classification of dental radiography without precise age information using convolutional neural networks. Healthcare. (2023) 11(8):1068. 10.3390/healthcare11081068 37107902 PMC10137502

[16] Zhang J Yu X Lei X Wu C. A novel deep LeNet-5 convolutional neural network model for image recognition. Comput Sci Inf Syst. (2022) 19(3):1463–80. 10.2298/CSIS220120036Z

[17] Rani S Ghai D Kumar S Kantipudi MP Alharbi AH Ullah MA. Efficient 3D AlexNet architecture for object recognition using syntactic patterns from medical images. Comput Intell Neurosci. (2022) 2022(1):7882924. 10.1155/2022/7882924 35634047 PMC9142332

[18] Liu F Gao L Wan J Lyu Z-L Huang Y-Y Han M. Recognition of digital dental x-ray images using a convolutional neural network. J Digit Imaging. (2023) 36(1):73–9. 10.1007/s10278-022-00694-9 36109403 PMC9984574

[19] Cejudo JE Chaurasia A Feldberg B Krois J Schwendicke F. Classification of dental radiographs using deep learning. J Clin Med. (2021) 10(7):1496. 10.3390/jcm10071496 33916800 PMC8038360

[20] Alsakar YM Elazab N Nader N Mohamed W Ezzat M Elmogy M. Multi-label dental disorder diagnosis based on MobileNetV2 and swin transformer using bagging ensemble classifier. Sci Rep. (2024) 14(1):25193. 10.1038/s41598-024-73297-9 39448640 PMC11502688

[21] Wang Q Zhao Y Zhang Z. Convolutional neural network-based multi-scale semantic segmentation for two-dimensional panoramic x-rays of teeth. In: Wang Y Chen X Qian D Ye F Wang S Zhang H, editors. MICCAI Challenge on Semi-Supervised Tooth Segmentation. Cham: Springer Nature Switzerland (2023). p. 1–13.

[22] Chlap P Min H Vandenberg N Dowling J Holloway L Haworth A. A review of medical image data augmentation techniques for deep learning applications. J Med Imaging Radiat Oncol. (2021) 65(5):545–63. 10.1111/1754-9485.13261 34145766

[23] Kebaili A Lapuyade-Lahorgue J Ruan S. Deep learning approaches for data augmentation in medical imaging: a review. J Imaging. (2023) 9(4):81. 10.3390/jimaging9040081 37103232 PMC10144738

[24] Chicco D Warrens MJ Jurman G. The coefficient of determination R-squared is more informative than SMAPE, MAE, MAPE, MSE and RMSE in regression analysis evaluation. PeerJ Comput Sci. (2021) 7:e623. 10.7717/peerj-cs.623/supp-1 34307865 PMC8279135

[25] Akbari A Awais M Bashar M Kittler J. How does loss function affect generalization performance of deep learning? Application to human age estimation. International Conference on Machine Learning. PMLR (2021). p. 141–51

[26] Roy R Ghosh S Ghosh A. Clinical ultrasound image standardization using histogram specification. Comput Biol Med. (2020) 120:103746. 10.1016/j.compbiomed.2020.103746 32421650

[27] Sichen D Jiwen G Yu W Jia L. Progress in the study of four dental age inference methods in the age inference of children and adolescents. Int J Front Med. (2023) 5(3):39–45. 10.25236/IJFM.2023.050307

[28] Corradi F Pinchi V Barsanti I Manca R Garatti S. Optimal age classification of young individuals based on dental evidence in civil and criminal proceedings. Int J Leg Med. (2013) 127:1157–64. 10.1007/s00414-013-0919-3 24081283

[29] Esan TA Yengopal V Schepartz LA. The Demirjian versus the Willems method for dental age estimation in different populations: a meta-analysis of published studies. PLoS One. (2017) 12(11):e0186682. 10.1371/journal.pone.0186682 29117240 PMC5678786

[30] Xiaoli Y Chao J Wen P Wenming X Fang L Ning L Prevalence of psychiatric disorders among children and adolescents in northeast China. PLoS One. (2014) 9(10):e111223. 10.1371/journal.pone.0111223 25360718 PMC4215989

[31] Wang J Dou J Han J Li G Tao J. A population-based study to assess two convolutional neural networks for dental age estimation. BMC Oral Health. (2023) 23(1):109. 10.1186/s12903-023-02817-2 36803132 PMC9938587

[32] Sivri MB Taheri S Ercan RGK Kırzıoğlu Ercan RG Yağcı Ü Golrizkhatami Z. Dental age estimation: a comparative study of convolutional neural network and Demirjian’s method. J Forensic Leg Med. (2024) 103:102679. 10.1016/j.jflm.2024.102679 38537363

